# Chinese University EFL Teachers' Perceived Support, Innovation, and Teaching Satisfaction in Online Teaching Environments: The Mediation of Teaching Efficacy

**DOI:** 10.3389/fpsyg.2021.761106

**Published:** 2021-10-11

**Authors:** Jiying Han, Chao Gao, Jing Yang

**Affiliations:** ^1^School of Foreign Languages and Literature, Shandong University, Jinan, China; ^2^School of Foreign Languages, Shandong Women's University, Jinan, China; ^3^School of Foreign Languages, Chengdu Normal University, Chengdu, China

**Keywords:** teaching support, teaching efficacy, teacher innovation, teaching satisfaction, online teaching environments, university EFL teachers

## Abstract

This study investigated the relationships between university EFL teachers' perceived teaching support, teacher innovation, and teaching satisfaction in online teaching environments, especially the mediating role of teaching efficacy. The results of an online questionnaire survey with 473 university EFL teachers revealed that although online peer support did not directly make any difference to teacher innovation and teaching satisfaction, greater perceived support in the form of teaching resources and teaching autonomy improved university EFL teachers' online teaching satisfaction. Online teaching efficacy significantly mediated the relationships between teaching support and teacher innovation and satisfaction. The results offer significant implications for improving the effectiveness of EFL teaching and promoting university EFL teachers' innovation and satisfaction in online teaching environments.

## Introduction

The outbreak of the COVID-19 pandemic has created substantial challenges around the world, including in the economic, health, and educational sectors (Ozamiz-Etxebarria et al., 2020). To maintain the sustainability of teaching and learning in higher education, university teachers have been required to shift entirely from traditional face-to-face teaching to online teaching within a very short time frame (Wang et al., 2021). This abrupt shift has greatly affected the perceptions and beliefs of university teachers, who have experienced confusion about and perceived challenges from online teaching (Ozamiz-Etxebarria et al., 2020). This has especially been the case in the field of second-language teaching and learning, where a supportive and interactive learning environment is necessary (Al-Samiri, 2021). EFL teachers' perceptions of and beliefs about online environments are not only pivotal to their instructional practises and classroom behaviour but also closely associated with student achievement (Eslami and Fatahi, 2008). Thus, it is of great significance to understand and assess university EFL teachers' perceptions of the newly mandated online teaching environments, as this may be conducive to understanding what they have experienced during the COVID-19 pandemic and thus facilitate the improvement of their teaching practises and promote their professional development.

The topic of teachers' beliefs about and perceptions of their own teaching abilities, known as teaching efficacy, has attracted a great deal of attention from educational professionals and researchers (Tschannen-Moran and McMaster, 2009; Richter and Idleman, 2017). Teachers with a higher sense of efficacy tend to engage in innovative teaching to meet the unique needs of their students and to achieve satisfaction with their teaching (Lee and Ogawa, 2021). Although previous studies have emphasised the significance of teaching efficacy and its relations to teaching environments and performances at different educational levels, there still remains inconsistency in the relationships between these variables in different educational environments. As researchers have pointed out that online teaching places teachers in a more complex environment that is different from traditional face-to-face instructions, an examination of teaching efficacy and the related variables in online environments is needed (Corry and Stella, 2018; Liu et al., 2021). Therefore, this study aimed to clarify the relationships between Chinese EFL teachers' teaching efficacy, perceived teaching support, innovation, and teaching satisfaction in online teaching environments in higher education. The study would offer insights into the university EFL online teaching process, in addition to providing practical implications for enhancing EFL teachers' teaching effectiveness and satisfaction, and stimulating them to innovate in online teaching.

## Literature

### Teaching Support

Teaching support is a key element in facilitating teaching and learning (Han et al., 2018), and its importance has been discussed extensively. It has been conceptualised in various dimensions at different educational levels. In primary and secondary schools, teaching support entails teaching resources, school administrative support, peer support, parental support, and community support (Tschannen-Moran and Hoy, 2002). In higher education, teaching support can be understood according to a scale consisting of three dimensions: teaching resources, peer support, and administrative support; this scale has been developed to measure university teachers' perceived teaching support (Chang et al., 2009). Recently, this scale has been revised and validated in a series of empirical studies conducted with Chinese university teachers (Han et al., 2018, 2020b). Teaching resources, including the availability of facilities, training, and technology, provide a favourable working environment for teachers, in which they can obtain help and support from colleagues and administrators to deal with job demands and stresses (Han et al., 2020a). Research has suggested that teachers' perceived peer support, teaching resources, and teaching autonomy are significant contributors to their online teaching practises (Richter and Schuessler, 2019). Therefore, in this study, teaching resources, peer support, and teaching autonomy were selected as the major indicators of university teachers' perceived online teaching support.

Teaching support is closely associated with students' learning satisfaction and learning outcomes (Wang et al., 2021). Teachers with teaching autonomy are likely to foster their students' autonomy in class and to have the freedom to autonomously regulate their learning processes (Martinek et al., 2020). Moreover, teachers' perceived teaching support enhances their teaching efficacy and teaching outcomes in different educational settings. In online teaching environments, teachers with ongoing support have a greater sense of efficacy when designing and delivering online courses (Richter and Idleman, 2017; Chung and Chen, 2018), and they experience less stress and burnout (Barni et al., 2019) and feel more satisfied with their teaching (Stickney et al., 2019). Recent research has also revealed that the enhancement of the teaching efficacy and satisfaction of EFL teachers in online teaching environments largely derives from administrative and peer support (Lee and Ogawa, 2021).

### Teacher Innovation and Teaching Satisfaction

Innovation is defined as a new or improved product or process that is significantly different from previous iterations (OECD, 2018), and it is undoubtedly crucial for the survival, sustainability, and success of different organisations (Wan et al., 2021). In educational organisations, teacher innovation refers to the degree to which a teacher designs and implements new and unusual teaching activities, with innovative teaching techniques, and assigns unique homework to students (Fraser et al., 1986). It not only improves students' learning outcomes and satisfaction (Wang et al., 2021) but also contributes to teachers' teaching effectiveness, professional development, and even the advancement of the knowledge society (Thurlings et al., 2015). Systematic reviews have indicated that teacher innovation is closely associated with a variety of individual and environmental factors, such as teaching efficacy and teaching motivation (Cao et al., 2020), support from co-workers and supervisors (Binnewies and Gromer, 2012), resources and professional autonomy (Eteokleous, 2008).

Teaching satisfaction is the product of teachers' attitudinal and affective responses to the perceived relationship between what they expect from their teaching and what their teaching offers or entails (Ho and Au, 2006). Recognised as one of the five pillars of quality education, teaching satisfaction is directly related to students' learning and teachers' performance and retention (Stickney et al., 2019). Research has repeatedly shown that teaching satisfaction is significantly associated with teaching efficacy (Skaalvik and Skaalvik, 2014; Han et al., 2020a) and school climate, as determined by such factors as support from supervisors, peer collegiality, and autonomy (Ismayilova and Klassen, 2019; Han et al., 2020b). In online teaching environments, university teachers find the online teaching experience rewarding and beneficial for their professional development, and they feel satisfied when offered appropriate training and flexibility in their schedules (Stickney et al., 2019). A recent study conducted by Hampton et al. (2020) also indicated that university teachers with a strong sense of teaching efficacy were more satisfied with their teaching in online courses. However, in the field of EFL teaching and learning, much attention has been paid to students' learning process and outcomes (Wang et al., 2019), very little is known about EFL teachers' teaching innovation and satisfaction, especially in online teaching environments.

### Teaching Efficacy as a Mediator

Self-efficacy refers to individuals' confidence in their abilities to carry out different tasks to accomplish desired outcomes (Bandura, 1997). It makes a significant difference to individuals' behaviour, motivation, and success (Tschannen-Moran and McMaster, 2009). In educational settings, teaching efficacy is a teacher's conviction of his/her perceived ability to bring about positive change and the expected teaching outcomes of students' learning (Tschannen-Moran and Hoy, 2002). Highly efficacious teachers tend to exhibit greater levels of organisation, preparation, and engagement in teaching, and they are more open to new ideas and methods to meet the needs of their students (Tschannen-Moran and Hoy, 2002).

Over the past two decades, many studies have investigated the construct of teaching efficacy at different educational levels. In higher education, the teaching efficacy of university teachers is defined as a belief in their abilities in course design, instructional strategy, technology use, classroom management, interpersonal relations, and learning assessment (Chang et al., 2011). Chang et al. (2011) developed a six-dimensional scale to assess university teaching efficacy by integrating efficacy theory into university teachers' conceptions of teaching. This scale was revised by Han et al. (2018) and validated with good psychometric features in studies conducted with Chinese university teachers in traditional face-to-face teaching environments (Han et al., 2018, 2020a). In comparison with traditional teaching, online teaching puts greater demands on teachers in areas such as effective teaching strategies, detailed course design, and better class management (Dykman and Davis, 2008). Thus, in accordance with the needs of online teaching environments, efficacy in course design, instructional strategy, and classroom management were selected as the indicators of university teachers' online teaching efficacy in this study.

Bandura (1997) postulated four principal sources for the sense of self-efficacy: mastery experience, vicarious experience, verbal persuasion, and physiological arousal. Empirical research has indicated that each of these four sources may enhance teaching efficacy in classrooms (Tschannen-Moran and McMaster, 2009). As teaching efficacy is context-specific, a number of contextual factors have been found to account for it, such as the availability of teaching resources, teaching constraints and physical space limitations, and school climate (Tschannen-Moran and McMaster, 2009). Previous studies have revealed that resources in the form of feedback and peer support may enhance teaching efficacy in higher education (Chang et al., 2011; Han et al., 2018). In online teaching environments, resources, training, and support structures are important determinants of teaching efficacy (Richter and Idleman, 2017; Corry and Stella, 2018).

Studies have demonstrated that teaching efficacy is strongly associated with teacher innovation, burnout, and job satisfaction in both traditional face-to-face teaching (Skaalvik and Skaalvik, 2014; Emin Türkoglu et al., 2017) and online teaching environments (Stickney et al., 2019; Hampton et al., 2020). Teachers who are more confident in their teaching abilities may face fewer difficulties in handling student misbehaviour, tend to use innovative teaching strategies (Jiang et al., 2021), and may experience lower levels of work pressure and higher levels of job satisfaction (Barni et al., 2019). Research has also emphasised the mediating effect of teaching efficacy on the relationship between job characteristics (e.g., job resources and job demand) and teacher well-being (e.g., teacher engagement and burnout) in traditional face-to-face instruction (Han et al., 2020a).

Although EFL teachers were found highly efficacious in using different teaching strategies and organising various classroom activities (Choi and Lee, 2016), very little is known about their teaching efficacy and related psychological factors in online teaching environments (Liu et al., 2021). Based on the aforementioned literature, the following hypotheses were established.

H1. Teaching resources is positively related to teacher innovation (H1a) and teaching satisfaction (H1b).
H2. Peer support is positively related to teacher innovation (H2a) and teaching satisfaction (H2b).
H3. Teaching autonomy is positively related to teacher innovation (H3a) and teaching satisfaction (H3b).
H4. Teaching resources (H4a), peer support (H4b), and teaching autonomy (H4c) are positively related to teaching efficacy.
H5. Teaching efficacy is positively related to teacher innovation (H5a) and teaching satisfaction (H5b).
H6. Teaching efficacy mediates the effect of teaching resources on teacher innovation (H6a) and teaching satisfaction (H6b).
H7. Teaching efficacy mediates the effect of peer support on teacher innovation (H7a) and teaching satisfaction (H7b).
H8. Teaching efficacy mediates the effect of teaching autonomy on teacher innovation (H8a) and teaching satisfaction (H8b).

**Figure 1:**
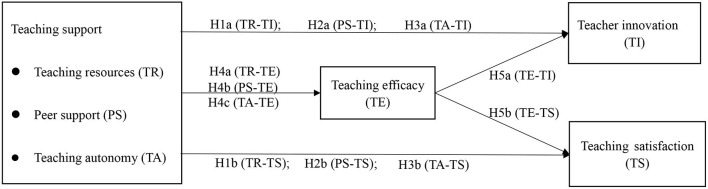
The hypothesised model.

Figure 1 shows the hypothesised model of this study.

## Methods

### Participants

The online questionnaire survey was conducted on a voluntary and anonymous basis in July 2020 after one full semester of online teaching. All participants were provided with a clear instruction of and well informed by the aim and process of the survey. Our sample consisted of 473 EFL teachers from 21 higher education institutions (HEIs) in Shandong, a province in Eastern China. Slightly more than half of the participants (55%) were female. Regarding professional rank, 3.6% were teaching assistants (the beginning rank of HEIs in China), 34.0% were lecturers, 46.5% were associate professors, and 15.9% were professors. Of the participants, 83.5% did not have online teaching experience before the outbreak of the COVID-19 pandemic.

### Instruments

The online questionnaire consisted of two parts. The first part was designed to collect the demographic information of the participants, such as gender, professional rank, and past online teaching experience. The second part was composed of four scales to assess university EFL teachers' perceived teaching support, teaching efficacy, teacher innovation, and teaching satisfaction in online teaching environments. All of the items were scored on a five-point Likert scale ranging from 1 (strongly disagree) to 5 (strongly agree).

### Teaching Support

The university EFL teachers' perceived online teaching support was assessed by nine items in three subscales. Online teaching resources and peer support were adapted from the revised 9-item Faculty-Perceived Teaching Support Scale (Han et al., 2018), and teaching autonomy was adapted from the Classroom Environment Scale (Bliuc et al., 2010). Some adaptations were made in consideration of the online teaching environments. Example items are ‘The university provides mentoring, training, and resources for online teaching” (teaching resources), “Colleagues offer advice on online teaching” (peer support), and “I can decide on specific ways and means to complete online teaching tasks” (teaching autonomy).

### Teaching Efficacy

Three dimensions adapted from the Faculty Teaching Efficacy Scale (Chang et al., 2009) were used to measure university EFL teachers' online teaching efficacy. Items were adapted in accordance with the online teaching environments: course design (five items, e.g., “I have sufficient ability to teach my courses online”), instructional strategy (five items, e.g., “I can teach online according to the level of students”), and classroom management (five items, e.g., “I can motivate students to participate in online learning”).

### Teacher Innovation

Four items were adapted from the College and university Classroom Environment Inventory (Fraser et al., 1986) to measure university teachers' perceived teacher innovation in online teaching environments. An example item is “I adopted a new teaching method different from those in face-to-face courses.”

### Teaching Satisfaction

University EFL teachers' perceived online teaching satisfaction was measured using a 5-item scale adapted from Ho and Au (2006). The items were slightly changed to adapt to online teaching environments. Examples items are “Generally, I am satisfied with my online teaching” and “I felt I achieved the online teaching objectives.”

### Data Analyses

Confirmatory factor analysis (CFA) was conducted using Mplus 8.3 to examine the validity of the four scales used in this study. Cronbach's α coefficients were computed to examine the reliability of the subscales using SPSS 25.0. Descriptive statistics (*M* and *SD*) and Pearson correlations were calculated for all factors. A full structural equation modelling (SEM) with a mediation analysis based on a bootstrapping strategy was then structured using Mplus 8.3 to identify the relationships between university EFL teachers' perceived teaching support, teaching efficacy, teacher innovation, and teaching satisfaction in online teaching environments. The goodness-of-fit indices used were χ^2^ statistics, degrees of freedom (*df*), comparative fit index (CFI), Tracker–Lewis index (TLI), and root-mean-square error of approximation (RMSEA). Additionally, Akaike Information Criterion (AIC) and Bayesian Information Criterion (BIC) were used for different model comparisons (the lower values of AIC and BIC are, the better the model fits) (van de Schoot et al. 2012). According to the literature, the acceptable model fit has CFI and TLI values of no <0.90 and an RMSEA value of no more than 0.10 (Schermelleh-Engel et al., 2003). The following guidelines were used to interpret the effect size of the regression coefficients: small = 0.10 to <0.20, medium = 0.20 to <0.30, large = ≥ 0.30 (Gignac and Szodorai, 2016).

## Results

### Validity and Reliability

CFA using Mplus 8.3 was conducted to test the validity of the three-factor measurement model of university EFL teachers' perceived online teaching support. The results, which are presented in Table 1, showed good model fit (χ^2^ = 83.77, *df* = 24, *p* < 0.001, CFI = 0.98, TLI = 0.98, RMSEA = 0.073), with the factor loadings ranging from 0.75 to 0.95. The Cronbach's α coefficients of the three subscales (see Table 2) were 0.89 (teaching resources), 0.93 (peer support), and 0.94 (teaching autonomy); being higher than 0.70, these scores indicated good internal consistency for each subscale.

**Table 1:** CFA results for the scales (*N* = 473).

**Scale**	**χ^2^**	***df***	***p***	**CFI**	**TLI**	**RMSEA**	**AIC**	**BIC**
Teaching support	83.77	24	0.00	0.98	0.98	0.073	–	–
Teaching efficacy								
First-order 3-factor	379.76	82	0.00	0.96	0.95	0.088	9656.97	9877.40
Second-order	304.96	80	0.00	0.97	0.96	0.077	9586.17	9814.92
Teacher innovation	6.56	2	0.00	0.996	0.99	0.069	–	–
Teaching satisfaction	3.71	1	0.00	0.999	0.99	0.076	–	–

**Table 2:** Reliabilities, descriptive statistics, correlations, and factor loadings.

	**1**	**2**	**3**	**4**	**5**	**6**
1. Teaching resources	(0.89)					
2. Peer support	0.57^**^	(0.93)				
3. Teaching autonomy	0.55^**^	0.57^**^	(0.94)			
4. Teaching efficacy	0.56^**^	0.52^**^	0.67^**^	(0.97)		
5. Teacher innovation	0.51^**^	0.51^**^	0.65^**^	0.72^**^	(0.93)	
6. Teaching satisfaction	0.56^**^	0.48^**^	0.66^**^	0.76^**^	0.62^**^	(0.94)
*Mean*	3.88	3.97	4.18	4.07	4.08	4.01
*Standard Deviation*	0.78	0.66	0.60	0.63	0.68	0.69
*Factor loadings*	0.75–0.95			0.67–0.92	0.80–0.93	0.82–0.97

** *p < 0.01; Cronbach's α coefficients in parentheses along the diagonal*.

The initial CFA results of the 15-item Teaching Efficacy Scale indicated that the first-order three-factor model fit the data well (χ^2^ = 379.76, *df* = 82, *p* < 0.001, CFI = 0.96, TLI = 0.95, RMSEA = 0.088). The factor loadings of items ranged from 0.67 to 0.92. However, the correlations between the three factors were very high: 0.77 between course design and classroom management, 0.81 between course design and instructional strategy, and 0.98 between instructional strategy and classroom management. As high correlations implied content overlap between factors, the three subscales were combined into a single composite factor to prevent multicollinearity. The results of the second-order CFA indicated that this model fitted the data well (χ^2^ = 304.96, *df* = 80, *p* < 0.001, CFI = 0.97, TLI = 0.96, RMSEA = 0.077) and was better than the first-order three-factor model (AIC = 9,586.17, BIC = 9,814.92). The Cronbach's α coefficient of the composite factor was 0.97.

The CFA results of the 4-item Teacher Innovation scale showed good model fit (χ^2^ = 6.56, *df* = 2, *p* < 0.001, CFI = 0.996, TLI = 0.99, RMSEA = 0.069), and the factor loadings of the items ranged from 0.80 to 0.93. The Cronbach's α coefficient was 0.93.

The construct validity of the 5-item scale of Teaching Satisfaction also indicated good model fit (χ^2^ = 3.71, *df* = 1, *p* < 0.001, CFI = 0.999, TLI = 0.99, RMSEA = 0.076). The factor loadings ranged from 0.82 to 0.97, and the Cronbach's α coefficient was 0.94.

### Descriptive Statistics and Correlations

Table 2 summarises the descriptive statistics and the correlation matrix. The mean scores of all of the factors were higher than the average of the 5-point Likert scale. Of the three subscales of online teaching support, teaching autonomy (*M* = 4.18, *SD* = 0.60) scored the highest, followed by peer support (*M* = 3.97, *SD* = 0.66) and teaching resources (*M* = 3.88, *SD* = 0.78). The correlation matrix indicated that all of the variables exhibited positive correlations between each other, with large effect sizes ranging from *r* = 0.48 to 0.76.

### SEM Analysis

**Figure 2:**
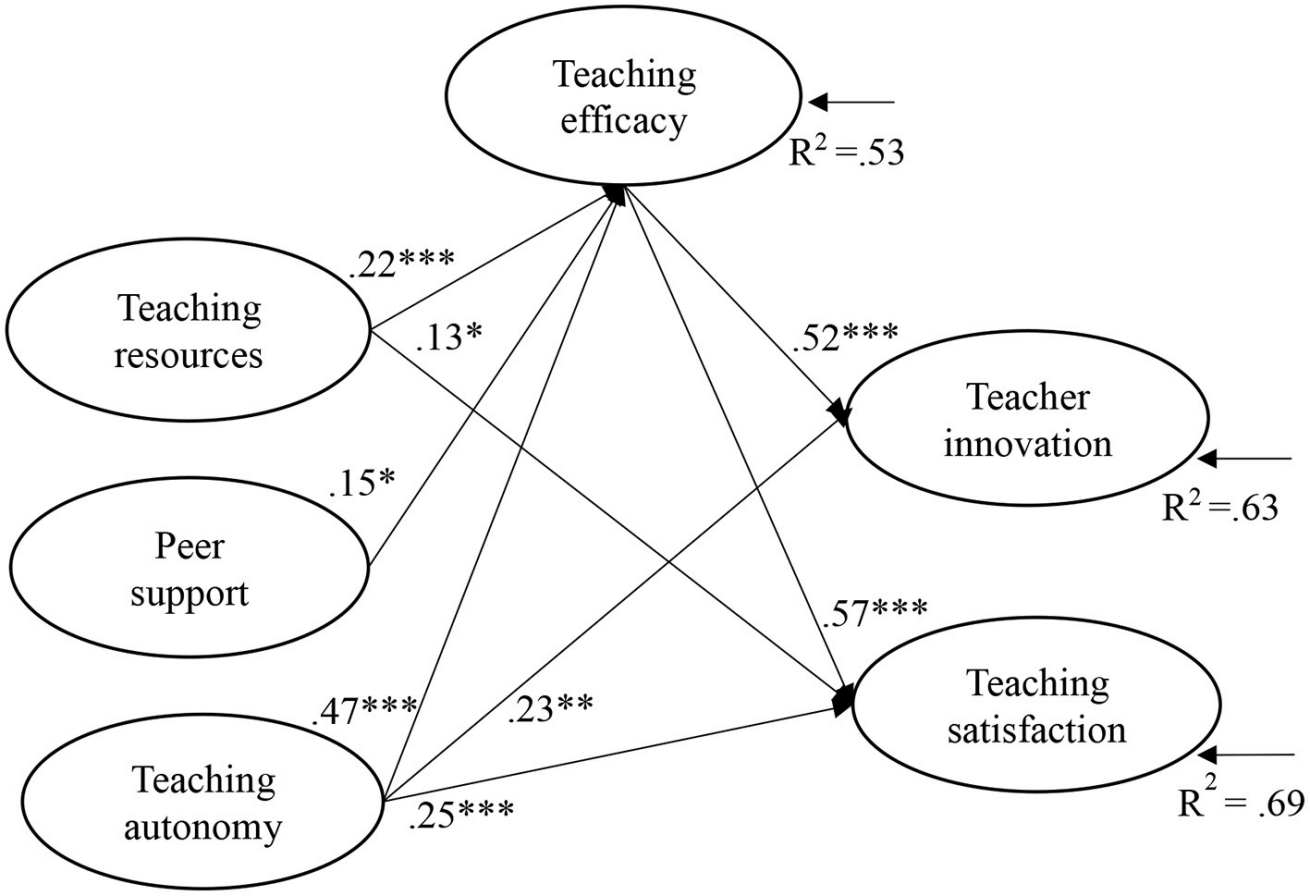
SEM model results showing significant regression path (*N* = 473). *p < 0.05, **p < 0.01, ***p < 0.001; Goodness-of-fit indices: χ^2^ = 1542.79, *df* = 446, *p* < 0.001, CFI = 0.93, TLI = 0.92, RMSEA = 0.072.

SEM was performed using Mplus 8.3 to test the hypotheses in the present study, exploring the relationships between university EFL teachers' perceived teaching support, teaching efficacy, teacher innovation, and teaching satisfaction in online teaching environments. In the hypothesised model, teaching support (teaching resources, peer support, and teaching autonomy) was the independent variable, teacher innovation and teaching satisfaction were the dependent variables, and teaching efficacy was the mediator. The SEM results presented in Figure 2 indicated that the data fit of the model was acceptable (χ^2^ = 1,542.79, *df* = 446, *p* < 0.001, CFI = 0.93, TLI = 0.92, RMSEA = 0.072), explaining the variances of 0.53 (teaching efficacy), 0.63 (teacher innovation), and 0.69 (teaching satisfaction).

Teaching autonomy was positively related to teacher innovation (β = 0.23, *p* < 0.01) and teaching satisfaction (β = 0.25, *p* < 0.001), with moderate effect sizes. Thus, H3a and H3b were supported. Teaching resources exhibited positive associations with teaching satisfaction (β = 0.13, *p* < 0.05), with a small effect size, supporting H1b. No significant relationships were found between teaching resources and teacher innovation, or between peer support and the dependent variables (teacher innovation and teaching satisfaction). Thus, H1a, H2a, and H2b were rejected.

Additionally, teaching support in the form of teaching resources (β = 0.22, *p* < 0.001), peer support (β = 0.15, *p* < 0.05), and teaching autonomy *(*β = 0.47, *p* < 0.001) were positively related to teaching efficacy, supporting H4a, H4b, and H4c. Teaching efficacy was positively related to teacher innovation (β = 0.52, *p* < 0.001) and teaching satisfaction (β = 0.57, *p* < 0.001), with large effect sizes. Thus, H5a and H5b were supported.

### Mediation Analysis

The mediation analysis was based on 5,000 bootstrapping samples with Mplus 8.3 to estimate the mediation effect of university EFL teachers' teaching efficacy on the relationship between teaching support and the dependent variables (teacher innovation and teaching satisfaction) in online teaching environments. In this study, the point estimate of the indirect effect was used to measure the effect size of the mediator (teaching efficacy). As suggested by Hayes (2009), an indirect effect size is significant if the lower bound and the upper bound of the 95% confidence interval does not include zero. The results summarised in Table 3 revealed that teaching efficacy significantly mediated the relationships between teaching support and the dependent variables (teacher innovation and teaching satisfaction) in online teaching environments. Specifically, the mediation effects of teaching efficacy between teaching resources and teacher innovation and teaching satisfaction showed small effect sizes (<0.2), supporting H6a and H6b; the mediation effects of teaching efficacy between peer support and teacher innovation and teaching satisfaction showed very small effect sizes (<0.1), supporting H7a and H7b; the mediation effects of teaching efficacy between teaching autonomy and teacher innovation and teaching satisfaction showed medium effect sizes (<0.3), supporting H8a and H8b.

**Table 3:** The estimate of direct and indirect effects of the 95% confidence intervals.

**Dependent variable**	**Independent variable**	**Direct effect**	**Indirect effect**	**95% confidence intervals**		**R^**2**^**
				**Lower 2.5%**	**Upper 2.5%**	
Teacher innovation	Teaching resources	0.07	**0.11**	0.05	0.21	0.63
	Peer support	0.07	**0.08**	0.02	0.16	
	Teaching autonomy	0.23	**0.24**	0.15	0.37	
Teaching satisfaction	Teaching resources	0.13	**0.12**	0.06	0.21	0.69
	Peer support	−0.02	**0.09**	0.02	0.16	
	Teaching autonomy	0.25	**0.27**	0.19	0.38	

*Items in bold indicating significant mediation effect*.

## Discussion

Results of SEM analysis with Mplus 8.3 revealed that teaching resources was positively related to teaching satisfaction, teaching autonomy was positively related to teacher innovation and satisfaction, teaching efficacy was positively related to all factors of teaching support, teacher innovation and teaching satisfaction. Mediation analysis based on 5,000 bootstrapping samples revealed that teaching efficacy significantly mediated the relationships between teaching support and the dependent variables (teacher innovation and teaching satisfaction) in online teaching environments. This study contributes to the knowledge on the perceptions and beliefs of university EFL teachers in online teaching environments by revealing the relationships between their perceived teaching support, teacher innovation, and teaching satisfaction, especially the mediating effects of teaching efficacy between teachers' perceived teaching support and teacher innovation and teaching satisfaction. The findings of this study offer insights into the EFL teaching process especially in online teaching environments and have significant implications for stimulating teacher innovation and enhancing teaching effectiveness in other EFL contexts.

### The Relationships Between Teaching Support, Teacher Innovation, and Teaching Satisfaction

The observed positive relationship between teaching resources and teaching satisfaction indicated that greater perceived support in the form of teaching resources increased the satisfaction of university EFL teachers in online teaching environments. Our result was consistent with the findings of previous research (e.g., Skaalvik and Skaalvik, 2017; Stickney et al., 2019), which also demonstrated a positive association between teaching resources and teaching satisfaction in both traditional and online teaching environments. Under the framework of the job demands-resources model, Skaalvik and Skaalvik (2017) found that different dimensions of teaching resources may well predict senior higher school teachers' satisfaction in traditional face-to-face instruction. Whereas Stickney et al. (2019) indicated that teachers' satisfaction in online higher education to a large extent was associated with teaching resources offered to them. Empirical studies have indicated that teachers who have access to sufficient teaching resources may become more dedicated to and vigorous in their teaching and thus feel more satisfied (Skaalvik and Skaalvik, 2017; Han et al., 2020b). Therefore, with more than 30 different online teaching platforms and numerous instructional programmes and training provided by Chinese governments and administrators (Zhou and Li, 2020), Chinese university EFL teachers may use these resources to meet the higher demands of online teaching, be more enthusiastic about and absorbed in their online teaching tasks, and feel more content with their accomplishments in online teaching.

However, the insignificant relationship between teaching resources and teacher innovation indicated that the availability of online teaching resources may not stimulate EFL teachers to innovate in their online teaching. This surprising result was inconsistent with previous research, which revealed a positive relationship between teaching resources and teacher innovation in schools in both traditional and online teaching environments (Eteokleous, 2008; Thurlings et al., 2015). As innovation is a complex multiple-stage process that involves idea exploration, idea generation, championing, and implementation and requires greater creative support (Kleysen and Street, 2001; Widodo and Gustari, 2020), it is challenging to stimulate teachers' willingness to teach innovatively in higher education (Cao et al., 2020). This is especially the case for university EFL teachers, most of whom do not have online teaching experience (83.5% in our study); thus, they may feel exhausted, less innovative, and overloaded when required to deliver online courses with very little time to prepare and to select suitable teaching platforms and teaching methods from an overwhelming supply of available teaching resources. Hence, teachers' innovative teaching may not rely on teaching resources offered to them in online teaching environments.

Unlike previous studies that indicated support from co-workers promoted teachers' idea generation and implementation (Binnewies and Gromer, 2012) and contentment with teaching (Ismayilova and Klassen, 2019) in traditional face-to-face instruction, our study unanticipatedly found that no significant relationships existed between peer support and teacher innovation and teaching satisfaction, suggesting that the improvement of innovative teaching and teaching satisfaction among university EFL teachers did not derive from the support of colleagues in online teaching environments. Empirical research has shown that in online teaching environments, although university teachers require greater support related to course content to complete online teaching tasks (Dykman and Davis, 2008), colleagues tend to offer forms of support that are more emotional and evaluative (Chung and Chen, 2018). Accordingly, the online support offered by colleagues may not be of the sort that teachers need in online teaching, and may even be irrelevant to teachers' innovative teaching and satisfaction.

Echoing with the findings of prior studies that found teachers' autonomy over their teaching conducive to the enhancement of innovative teaching (Thurlings et al., 2015; Cao et al., 2020) and satisfaction (Stickney et al., 2019), we also found that teaching autonomy was positively related to teacher innovation and teaching satisfaction, indicating that university EFL teachers' perceived autonomy in online teaching facilitates their teaching innovation and satisfaction. Empirical research has indicated that autonomy is highly conducive to creativity insofar as it provides a sense of freedom, responsibility, and control over work outcomes, making the job more exciting (Coelho and Augusto, 2010; Lu et al., 2012). When university EFL teachers perceive more autonomy in online teaching, they may feel both freedom and a responsibility to try new ideas, decide upon and select their teaching approaches, design teaching tasks, and evaluate students' learning (Esfandiari and Kamali, 2016), and they will consequently feel greater satisfaction with their teaching outcomes (Hampton et al., 2020).

### Teaching Efficacy as a Mediator of the Relationships Between Teaching Support and Teacher Innovation and Teaching Satisfaction

The SEM results indicated that university EFL teachers' perceived teaching efficacy was positively related to teaching support, teacher innovation, and teaching satisfaction in online learning environments. Although research on the relationships between these variables for university EFL teachers is scarce in online teaching environments, evidence from previous studies indicates that teachers' perceived teaching support enhances their teaching efficacy in both traditional face-to-face instruction (Chang et al., 2009; Han et al., 2018) and online teaching environments (Chung and Chen, 2018; Richter and Schuessler, 2019). As efficacy is context-specific, teachers make efficacy judgements partly by assessing the resources and constraints available to them in specific teaching contexts (Tschannen-Moran and Hoy, 2002); accordingly, more resources, flexibility in scheduling, and autonomy over their teaching may promote teachers' confidence in their ability to teach. Additionally, peer support, serving as verbal persuasion, is a major source of teaching efficacy (Bandura, 1997). Empirical studies have shown that the greater the perceived support from colleagues, the more confident teachers feel in their teaching abilities (Tschannen-Moran and Hoy, 2002; Han et al., 2018). Therefore, teachers' perceived support in the form of teaching resources, peer support, and teaching autonomy strongly contributes to the enhancement of their online teaching efficacy.

The results of this study indicated positive relationships between teaching efficacy and teacher innovation and teaching satisfaction in online teaching environments. This suggests that university EFL teachers with greater confidence in their online teaching abilities are more likely to perceive innovation in their work and be satisfied with their teaching process and outcomes. Empirical studies have demonstrated that highly efficacious teachers tend to exhibit greater levels of organisation, preparation, and engagement in teaching, and are more open to and implement new ideas and methods to meet the needs of their students (Tschannen-Moran and Hoy, 2002). Moreover, teachers who are more confident in their ability to teach may have strong communication in their workplaces (Caprara et al., 2006; Emin Türkoglu et al., 2017), face fewer difficulties in handling student misbehaviour, and experience lower levels of work pressure and higher levels of job satisfaction (Barni et al., 2019; Hampton et al., 2020). Thus, increases in EFL teachers' confidence in their teaching may lead to increases in their levels of innovation and satisfaction with their teaching.

Our results also indicated that the significant mediating role of online teaching efficacy increased the explanatory power of university EFL teachers' perceived online teaching support. Specifically, the mediation analysis revealed that the effect sizes of teaching efficacy as a mediator scored the highest on the effect of teaching autonomy than that on teaching resources and peer support, indicating that teaching autonomy has the strongest power to predict university EFL teachers' teaching efficacy towards enhanced innovation and satisfaction. And for university EFL teachers who perceived autonomy in online teaching, the effect of their perceived support on teacher innovation and teaching satisfaction was greatly actualised through their increased level of teaching efficacy. Additionally, the very small mediating effect sizes of teaching efficacy on the effect of peer support on teacher innovation and teaching satisfaction revealed that the increased teaching efficacy had no practical meanings for the relationships between these variables. Although no direct relation existed between teaching resources and teacher innovation, teaching efficacy was found a significant mediator for the effect of teaching resources on teacher innovation. This indicated that with the increased level of teaching efficacy, EFL teachers who perceived more teaching resources tend to innovate in their online teaching activities. Empirical studies have shown that teaching efficacy is conducive to teachers' online innovative teaching (Yu et al., 2021). Theoretically, teachers who are confident in their teaching capabilities tend to make full use of available resources to try out new ideas and teaching methods to meet their students' needs (Tschannen-Moran and Hoy, 2002). Therefore, without a direct effect on teacher innovation, the effect of university EFL teachers' perceived teaching resources was mainly actualised through the mediation of teaching efficacy.

### Limitations and Future Direction for Research

This study has yielded some preliminary findings on Chinese university EFL teachers' perceptions and beliefs in online teaching environments. It also has some limitations, which may be noted as directions for future research. First, this cross-sectional study was insufficient to confirm causal relationships between university EFL teachers' perceived teaching support and their teaching efficacy, teacher innovation, and teaching satisfaction; thus, a longitudinal research design may help to clarify the directionality of the regression paths in future research. Second, the results of this study were based on teachers' self-reported measurements, and these reports may exaggerate or underreport the target perceptions for different reasons. Future research could collect more objective data using multiple research methods, such as a mixed-methods design. Thirdly, the hypothesised relationships between teaching support, teaching efficacy, teacher innovation, and teaching satisfaction in the present study are just one of the possibilities based on the review of the literature. There might be other possibilities of their relations, and this would be one of the directions for future research.

## Implications for Practise

This study investigated the relationships between Chinese university EFL teachers' perceptions of teaching support, teaching efficacy, teacher innovation, and teaching satisfaction in online teaching environments. The results offer practical implications for improving the effectiveness of teaching and promoting university EFL teachers' innovation and teaching satisfaction in online teaching environments.

The relationships between university EFL teachers' perception of teaching support and teacher innovation and teaching satisfaction highlight the important role of teaching autonomy in facilitating desirable performance in online teaching environments. Accordingly, teachers may be granted more freedom and autonomy over their online teaching practises, with which they might make their online teaching more effective, innovative, and satisfying. For example, they may be allowed to autonomously design their online classes with abundant teaching materials, deliver online courses on their preferred teaching platforms, select suitable instructional strategies to complete their online teaching tasks, and evaluate their students with appropriate standards. Meanwhile, university administrators may offer teachers extra teaching resources, which may be helpful in the creation and implementation of new ideas and teaching procedures, such as training and programmes pertinent to creative instructional strategies and course design. In addition, colleagues could be guided to offer creative support specifically related to teaching content and knowledge transmission, which might improve teachers' innovation and satisfaction.

Considering the relationships between teacher innovation and other variables, the promotion of university EFL teachers' innovative teaching in online environments may stem directly from teachers' autonomy over their teaching practises. The significant mediation effects of teaching efficacy highlight the need of EFL teachers' improved teaching confidence in stimulating innovative teaching when they were provided with adequate teaching resources (i.e., training and lectures on creative teaching strategies) and creative peer support in online teaching environments. Therefore, university administrators may provide a wide range of online teaching support, such as the increased availability of online teaching resources and autonomy, as well as the promotion of peer support; they may also plan and implement effective training programmes to help teachers improve their confidence in completing online teaching tasks. Additionally, university EFL teachers may be guided to utilise such support to innovate in online teaching. For example, they may consider re-designing and implementing online teaching activities, and adopting innovative instructional strategies different from face-to-face instruction, to sustain students' attention and nurture a pleasant online learning environment.

## Data Availability Statement

The raw data supporting the conclusions of this article will be made available by the authors, without undue reservation.

## Ethics Statement

The studies involving human participants were reviewed and approved by Shandong University. The patients/participants provided their written informed consent to participate in this study.

## Author Contributions

JH and CG wrote and refined the article. JY was involved in data collection and analysis. All authors contributed to the article and approved the submitted version.

## Funding

This work was supported by Humanity and Social Science Fund of China's Ministry of Education under Grant Number 21YJA880017 and Young Scholars Programme of Shandong University under Grant Number 2017WLJH09.

## Conflict of Interest

The authors declare that the research was conducted in the absence of any commercial or financial relationships that could be construed as a potential conflict of interest.

## Publisher's Note

All claims expressed in this article are solely those of the authors and do not necessarily represent those of their affiliated organizations, or those of the publisher, the editors and the reviewers. Any product that may be evaluated in this article, or claim that may be made by its manufacturer, is not guaranteed or endorsed by the publisher.
