# Supplementary material for: Chinese University EFL Teachers' Perceived Support, Innovation, and Teaching Satisfaction in Online Teaching Environments: The Mediation of Teaching Efficacy
Source: Front Psychol. 2021 Oct 11;12:761106. doi: 10.3389/fpsyg.2021.761106 (PMC8542998; doi:10.3389/fpsyg.2021.761106)
Supplement: Supplementary file 1 [file Data_Sheet_1.docx]

# Appendix

***Teaching support***

*Teaching resources*

1. The university provides mentoring, training, and resources for online teaching.
2. The university provides the technology and software resources for online teaching.
3. The university provides hardware equipment required for online teaching (such as live classrooms, etc.).

*Peer support*

1. Colleagues offer advice on online teaching.
2. Colleagues encourage and support me if I have trouble with my online teaching.
3. Colleagues share online teaching experiences with me.

*Teaching autonomy*

1. I can decide on specific ways and means to complete online teaching tasks.
2. I can select a teaching procedure to complete the online teaching.
3. I can choose which method to use to complete my online teaching.

***Teaching efficacy***

*Course design*

1. I have sufficient ability to teach my courses online.

2. I can set up comprehensive online teaching objectives.

3. I can select appropriate online teaching material.

4. I can arrange an appropriate online timeline for the curricular progress.

5. I can prepare my online teaching material before class sessions.

*Instructional strategy*

1. I can teach online according to the level of students.

2. I can utilize effective online teaching methods to improve students’ grades.

3. I can modify my online teaching activities during class sessions to sustain students’ attention.

4. I have confidence in inspiring and maintaining students’ online learning motivation.

5. I can utilize various online inquiring skills to stimulate students’ higher-level thinking skills and discussions.

*Classroom management*

1. I can motivate students to participant in online learning.

2. I can nurture a pleasant online learning environment.

3. I can maintain a good relationship with my students through online teaching.

4. I can share my personal experiences online with students to promote emotional bonding.

5. I can listen to my students to understand their thoughts.

***Teacher innovation***

1. I adopt a new teaching method different from those in face-to-face courses.
2. I design new online learning activities for students to get involved.
3. I use various and innovative teaching methods in the online courses.
4. I assign different learning tasks in online courses.

***Teaching satisfaction***

1. Generally, I am satisfied with my online teaching.

2. I felt I achieved the online teaching objectives.

3. I am satisfied with the online teaching results.

4. I think online teaching is effective.

5. I felt comfortable in online teaching.
